# Neurological Outpatients Prefer EEG Home-Monitoring over Inpatient Monitoring—An Analysis Based on the UTAUT Model

**DOI:** 10.3390/ijerph192013202

**Published:** 2022-10-13

**Authors:** Ulrike Baum, Frauke Kühn, Marcel Lichters, Anne-Katrin Baum, Renate Deike, Hermann Hinrichs, Thomas Neumann

**Affiliations:** 1 Department of Neurology, Otto-von-Guericke University Magdeburg, Leipziger Str. 44, 39120 Magdeburg, Germany; 2 Institute for Sensory and Innovation Research (ISI GmbH), Ascherberg 2, 37124 Rosdorf, Germany; 3 Chair of Marketing and Retailing, Faculty of Economics and Business Administration, Chemnitz University of Technology, Reichenhainer Straße 39, 09126 Chemnitz, Germany; 4 Leibniz Institute for Neurobiology, Brenneckestraße 6, 39118 Magdeburg, Germany; 5 Center for Behavioral Brain Sciences (CBBS), Universitätsplatz 2, 39106 Magdeburg, Germany; 6 Chair in Health Services Research, School of Life Sciences, University of Siegen, Am Eichenhang 50, 57076 Siegen, Germany; 7 Chair in Empirical Economics, Otto-von-Guericke-University Magdeburg, Universitätsplatz 2, 39106 Magdeburg, Germany; 8 Research Campus STIMULATE, Otto-von-Guericke-University Magdeburg, Sandtorstraße 23, 39106 Magdeburg, Germany

**Keywords:** home monitoring, electroencephalography (EEG), mobile EEG, partial least squares structural equation modelling (PLS-SEM), UTAUT

## Abstract

Home monitoring examinations offer diagnostic and economic advantages compared to inpatient monitoring. In addition, these technical solutions support the preservation of health care in rural areas in the absence of local care providers. The acceptance of patients is crucial for the implementation of home monitoring concepts. The present research assesses the preference for a health service that is to be introduced, namely an EEG home-monitoring of neurological outpatients—using a mobile, dry-electrode EEG (electroencephalography) system—in comparison to the traditional long-time EEG examination in a hospital. Results of a representative study for Germany (*n* = 421) reveal a preference for home monitoring. Importantly, this preference is partially driven by a video explaining the home monitoring system. We subsequently analyzed factors that influence the behavioral intention (BI) to use the new EEG system, drawing on an extended Unified Theory of Acceptance and Use of Technology (UTAUT) model. The strongest positive predictor of BI is the belief that EEG home-monitoring will improve health quality, while computer anxiety and effort expectancy represent the strongest barriers. Furthermore, we find the UTAUT model’s behavioral intention construct to predict the patients’ decision for or against home monitoring more strongly than any other patient’s characteristic such as gender, health condition, or age, underlying the model’s usefulness.

## 1. Introduction

The German public health sector faces challenges, resulting from an increasingly aging population, accompanied by financial pressure and also physician shortages, especially in rural areas [1]. In this context, telemedicine is gaining importance for the capabilities it offers in the assessment and management of diseases across different medical specialties [2]. Furthermore, these developments have been pushed forward since the COVID-19 pandemic began in 2019 [3,4]. Beyond telephone and video consultation between physicians and patients as common telemedical implementations, home monitoring devices in combination with an appropriate infrastructure for safely transferring data–e.g., from rural areas to hospitals or doctors’ offices–offer promising results [5].

Electroencephalography (EEG) is an important diagnostic tool in the neurological sector. During an EEG examination, electrical fields, which are generated by the ongoing neural activity of the brain, are recorded at the scalp using appropriate electrodes and amplifiers. Clinically the EEG reflects a correlate of general brain function see [6,7]. Accordingly, it serves as a standard method to diagnose patients suffering from neurologic diseases, such as epilepsy, stroke, dementia, occasional unconsciousness, concussion, and others. Under routine conditions the EEG is recorded at 21 different electrode sites (i.e., 21 EEG-channels) over a period of approximately 20 min [8,9] in a doctor’s office or in a neurological hospital department. However, given that many diagnostic questions (for instance epilepsy) require much longer recordings [7,9,10,11,12,13,14,15,16,17,18,19] and hospitalization of patients is expensive [11,12,20], the idea of mobile EEG devices that can be used at home arose in the seventies with the development of ambulatory cassette EEG recorders [21,22]. While during the eighties and nineties the further development and digitalization of mobile EEG devices improved their medical and diagnostic use [23,24,25,26,27,28], home monitoring was still cumbersome as the EEG systems could only be placed and removed by medical staff who had to add electrode gel or conductive paste. Only the development of “dry electrodes” which do not require a proper preparation of the skin before recording [29] was the starting point to create EEG systems that allow for a user-friendly and autonomous use of patients. There are several modern mobile EEG systems, see [30] for an exemplary overview, and studies that compare mobile systems with conventional EEG systems in clinical environments [31,32,33,34]. However, there is a lack of studies with mobile, patient-controlled, and dry-electrode EEG systems that meet the medical requirements and are used for home monitoring.

Due to the mentioned difficulties regarding medical care in rural regions in Germany, EEG home-monitoring has been proposed as an alternative [35,36] to the conventional inpatient monitoring and has been the motivator for our *HOME* (**Ho**me-**M**onitoring and **E**ducation) project.

We created the *HOME* project in order to develop an EEG-based home-monitoring concept for patients with neurological disorders. This was achieved using the Fourier ONE^TM^ (F1) (Nielsen Tele Medical GmbH, Magdeburg; now TeleMedi GmbH, Magdeburg), a new mobile EEG system that meets the technical and practical requirements we considered essential for this purpose, e.g., CE certification, wireless connectivity, dry electrodes, comfort, portability, and patient friendliness [37]. To meet the key goals of the *HOME* project, we conducted several studies. In a first step, the HOME^ONE^ study confirmed the technical usability and efficacy of F1 when compared to conventional EEG systems under clinical conditions [38] and, in the second step, proved both the feasibility and diagnostic/therapeutic yield of EEG home-monitoring [39]. Additionally, the HOME^EPI^ study also confirmed the technical usability and efficacy of F1 when compared to conventional EEG systems but with a special focus that included only patients with suspected epilepsy [40]. Feedback regarding the comfort of F1 has been documented elsewhere [41].

In the case of home monitoring, patients must accept the burden of creating EEG recordings autonomously. As their personal commitment is essential for the implementation of this new medical care option, this led to the creation of our HOME^TA^ study, designed with the objective of finding the preferences of both patients and potential patients (defined as non-patients further on) when faced with either a long-term EEG examination in a hospital (inpatient examination) or EEG home-monitoring, including the predictors behind the preference.

Taking the assumption that the preference for using EEG home-monitoring may depend on the acceptance of a health service that includes the autonomous use of an EEG system, we decided to investigate this aspect in more detail. In this regard, we incorporated a technology acceptance model in our study. 

We opted for the Unified Theory of Acceptance and Use of Technology (UTAUT) model [42] which is based on the earlier Technology Acceptance Model (TAM) [43]. Both theories are widely used models in health technology acceptance studies according to several reviews [44,45]. Recent applications of the UTAUT model in the patients’ or potential patients’ acceptance area can be divided into two approaches: studies that (1) investigate the acceptance of telehealth services [46] or mobile health solutions [47,48,49] as a broad concept, and (2) refer to a specific service or product (e.g., apps) [50] or healthcare wearable devices [51,52]. To the best of our knowledge, there is no other study assessing preferences for an EEG home-monitoring based on patients’ acceptance regarding this specific health service. The HOME^TA^ study we report here can bridge this gap.

Our research is based on an extended version of the UTAUT model [46] which was originally designed to investigate the acceptance of home telehealth services in general. However, besides some necessary changes, it seemed promising to apply this approach to assess several drivers that may influence the behavioral intention to use an EEG home-monitoring.

## 2. Methods

### 2.1. Procedure

The study followed a 2 (participant type: patient vs. non-patient) × 2 (introductory video: yes vs. no) between-subject design. In total, 488 participants completed the survey (gross sample). We excluded all individuals who needed less than 10 min to do so (n=62) and all those who failed the attention check question (n=5), resulting in a net sample of n=421 participants with a mean age of 49.13 years and SD=14.62 (55% males). Table 1 presents the participants characteristics of the remaining 421 participants.

Participants were asked to imagine the following scenario: their neurologist recommends a long-term EEG investigation after they have collapsed in a garden (Appendix A). The medical check-up in question can be performed as either inpatient monitoring or home monitoring. Subsequently, we presented both options, each with a picture of the corresponding EEG device and a description (Appendix B). 

### 2.2. Recruitment

We used two different groups of participants for our study. First, a group of 40 randomly selected neurological patients of the University Hospital in Magdeburg (Germany) who already were confident with the home monitoring system. These participants were free to choose whether to fill-out the survey online (n=30) or offline (n=10). Inclusion criteria were a minimum age of 18 years. Second, a group of participants (non-patient) recruited by a professional German online panel provider (myonlinepanel GmbH), who also required a minimum age of 18 years as inclusion criteria. The selection of suitable participants from the online panel pool was based on an equal distribution of age, gender and living environment (urban or rural). Participants (n=381) recruited by the online panel provider did not know the EEG home-monitoring system. Additionally, we randomly allocated all online participants (i.e., patients and non-patients) to either the video condition or no-video condition. As a result, in the net sample (n=421), nearly half of participants (n=215) viewed a video covering the usage of an example mobile EEG device suitable for home monitoring, while in the no-video condition participants only received pictures and written descriptions of both options (n=206).

The resultant four groups (1) patient/video, (2) patient/no-video, (3) non-patient/video, and (4) non-patient/no-video did not differ significantly in terms of age, graduation, or vocational qualification level, smallest p=0.223, but did in gender: $\chi^{2}\left(6\right)=19.50$; p=0.003 (for details see Table 1). Please note that we obtained one divers observation, when excluding, we got $\chi^{2}\left(3\right)=3.71$; p=0.295. Thus, gender is not significantly different across the four groups. 

### 2.3. Measurement

After the introduction, participants indicated their preference between the two medical care variants via a Likert scale (‘Which of the two options would you prefer for a long-term EEG examination?’). The scale ranged from 1 = strong preference for inpatient monitoring to 7 = strong preference for home monitoring. This allowed us to obtain granular information on the merits of both medical care options. Furthermore, they filled out all the questions from the extended UTAUT model based on Cimperman et al. [46] for measuring the acceptance of EEG home-monitoring. This meant also incorporating 7-point Likert-scaled items ranging from 1 = totally disagree to 7 = totally agree. The model comprised the main UTAUT constructs of *performance expectancy* (PE), *effort expectancy* (EE), *social influence* (SI), and *facilitating conditions* (FC), which were extended by the constructs of *perceived security* (PS), *computer anxiety* (CA), and *doctor’s opinion influence* (DC) to identify individual drivers of *behavioral intention* to use home monitoring (BI). Appendix C presents all the measurement model details, including wording, translation, and validity assessment. For identifying the current health status, the German translation of the 36-item Short-Form Health Survey [53] was used, including for *physical functioning* (PF), role limitations due to *physical health* (PH), role limitations due to *emotional problems* (EP), *energy/fatigue* (EF), *emotional well-being* (EW), *social functioning* (SF), *pain* (P), *general health* (GH), and *health change* (HC). Appendix D presents construct wording and the reliability assessment. Finally, participants also provided socio-demographic information.

## 3. Results

### 3.1. Preference Analysis

Individuals report a higher relative preference for home monitoring compared to inpatient monitoring (mean=5.15, SD=2.01, one-sample t-test against the scale midpoint $t\left(420\right)=11.73,\;p<0.001$). Furthermore, no significant difference concerning preference is found to exist between patients and non-patients ($mean_{non-patient}=5.11,\;SD=2.01$, $mean_{patient}=5.55,\;SD=2.00$, $F\left(1,\;420\right)=1.78,\;p=0.183$). In line with our expectations we found that participants who saw the video reported a significantly higher relative preference for home monitoring ($mean_{video}=5.37,\;SD=1.83$, $mean_{no\;video}=4.91,\;SD=2.15$, $F\left(1,\;420\right)=5.57,\;p<0.001$), This result illustrates the need for a proper explanation of the examination options. However, no significant interaction emerges between the participant type (patient vs. non-patient) and the video factor ($F\left(1,\;420\right)=0.681,\;p=0.410$). Supporting the face validity of findings, a higher preference for home monitoring significantly correlates with individuals’ behavioral intention (BI) in the UTAUT model presented in the next section ($r=0.49,\;t\left(419\right)=11.51,\;p<0.001$) which is the case for the full sample as well as all four study groups separately. Based on these results, the next step focused on identifying drivers of participants’ BI to use home monitoring. Because our goal is to explain the BI construct’s variance rather than replicating perfectly the original variance covariance matrix, we opt for partial least squares structural equation modeling PLS-SEM [54,55,56]. This decision is in line with other authors in the field of research who usually apply PLS-SEM when implementing the UTAUT model [46,57,58,59,60], especially when their research is intended to predict and explain the key target construct or to identify the key construct’s main drivers [61,62].

### 3.2. Drivers of BI

**Figure 1:**
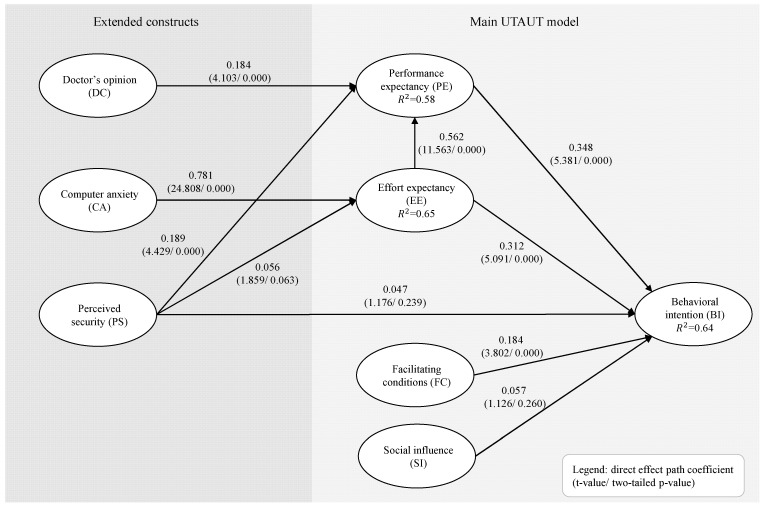
Estimated path model with direct effects (t-values/ *p*-values).

We set up the extended UTAUT model with SmartPLS 3.0 [63] (Figure 1). Due to the identical results of the preference analysis above, as well as the sample size of the patient group (n=40), we pooled the patient and non-patient groups. Please note that we also checked the results for differences between the groups (patient type and video factor) by means of a multi-group analysis. However, no significant differences emerged that limit our results’ interpretation.

The analysis starts with an evaluation of the measurement model’s reliability and validity for which Appendix C presents the details. Overall, the measurement model raises no reliability or validity concerns. The main analysis evaluates the structural model and is based on 5000 bootstraps and two-tailed *p*-values a(=5%). Multicollinearity is not a problem among exogenous constructs as the inner variance inflation factors (VIFs) are all < 2.5 (Appendix C). Figure 1 presents the model with the direct path coefficients, t-values, and *p*-values. Additionally, Table 2 provides the indirect and total effects, together with full statistics.

Our main interest is to understand which exogenous constructs explain the final endogenous construct BI. Results show that there are three significant direct drivers and three significant indirect drivers. PE has the strongest direct effect (b=0.348, p<0.001), followed by EE (b=0.312, p<0.001) and FC (b=0.184, p<0.001). Thus, participants’ belief in an improvement of health quality is the main driver for their BI to favor home monitoring. Additional drivers include participants’ belief in there being less effort required to use home monitoring systems (higher EE scores indicate less expected effort) and to obtain technical support (FC). SI does not have a significant effect on BI (b=0.057, p=0.260), which stresses that social influence (e.g., friends and colleagues) is not a BI driver. CA (higher scores indicate the absence of computer anxiety) has the strongest significant indirect effect on BI via EE (b=0.397, p<0.001). Certainty regarding the safety of personal health information (PS) and confidence in doctors’ expertise (DC) also have significant indirect effects on BI via PE (respectively, b=0.066, p=0.001 and b=0.065, p<0.001).

Next, we checked via a multigroup analysis [64] for differences between patients vs. non-patients as well as between the video vs. no-video groups. For the first factor, we find one significant effect of FC on BI ($b_{nonpatient-patient}=p=0.048$). Nevertheless, both groups provide a significant effect of FC on BI. For patients, however, the effect of ‘belief in receiving technical support when using home monitoring’ on their behavioral intention is even stronger. Moreover, no differences exist between the video and no-video conditions (smallest p=0.256).

**Figure 2:**
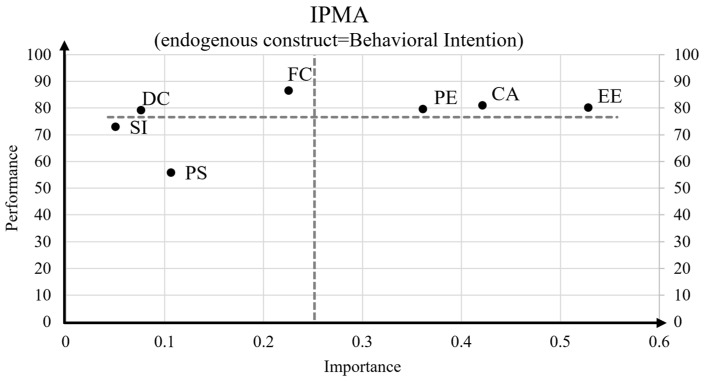
Estimated importance-performance map for the endogenous construct BI.

To summarize the exogenous constructs’ explanatory power for BI, we implemented an importance-performance map analysis (IPMA), which portrays the exogenous constructs’ importance (i.e., their total effects in explaining BI on the x-axis) as well as their rescaled latent construct scores on the y-axis that represents the status quo for a construct from worst–0, to best–100 [65]. Figure 2 provides the resultant IPMA.

The x-axis in Figure 2 visualizes that, in total (sum of direct and indirect effects), EE has the largest effect on BI, followed by CA and PE. In contrast, SI, DC, and PS have only marginal effects. The y-axis however underlines that all three above-average important constructs also have high and above-average latent construct scores (i.e., the y-axis highlights a construct’s status quo). Thus, there is only limited room for possible improvements. Keep in mind that EE and CA are negatively scaled, with high values representing the absence of computer anxiety as well as low effort expectations. In conclusion, although PS only has a marginal influence in explaining BI, there is much room for improving the current status quo when it comes to the perceived security of EEG home-monitoring.

Next, we assessed the predictive validity of the PLS-SEM model in terms of its ability to predict the endogenous constructs’ indicators by means of their exogenous constructs’ indicators as compared to a classical OLS regression. This procedure is called PLSpredict in the literature [66,67]. Since we handle mediating constructs in our structural model (e.g., EE), we applied the *direct antecedents* [68] approach drawing on the R package *seminr* [69]. Specifically, we used 10 folds with 10 replications, which results in the root mean squared errors of prediction (RMSE) in Table 3.

Results in Table 3 indicate that only the minority of prediction errors of the PLS-SEM model is lower as compared with the linear OLS model. Thus, following conventional thresholds, the predictive power has to be regarded as rather low [70]. Therefore, we take additional measures to evaluate PLS model’s abilities in predicting the respondents’ relative preference for EEG home-monitoring vs. its stationary counterpart in the next section.

### 3.3. Impact of BI Score, Health Measurement, Age, and Gender on Preference

Finally, we performed a regression analysis (OLS) to assure that BI is a real predictor for individuals’ preference between home monitoring and inpatient monitoring beyond control variables. For this purpose, we set up regression models with varying sets of predictors (Table 4). Model 1 utilizes solely the BI construct score from PLS-SEM, whereas Model 2 extends the model according to the participants’ health status. Afterward, Model 3 includes demographics. Lastly, Model 4 draws on all previous predictors along with the participant type (patient vs. non-patient) as well as the video factor (yes vs. no). For the health status, we obtained construct scores by simply averaging individual items, after reverse coding if necessary. The results point out a significant overall model for all four models, with Model 4 resulting in $F\left(14,\;405\right)=11.151$, p<0.001. Across all models, BI is a robust predictor of participants’ preferences. Model 4 indicates that the video factor is another significant predictor (b=0.476, t=2.772, p<0.006). Specifically, the preference for choosing home monitoring over inpatient monitoring increases when an introductory video is being watched. Nevertheless, a summary of Table 4 shows that the extension of Model 1 to Model 4 does not result in a significantly enhanced explanation of the dependent variable ($adjusted\;R_{Model\;1}^{2}=0.234$ vs. $adjusted\;R_{Model\;4}^{2}=0.253$. Consequently, BI from the extended UTAUT model seems to be the main driver for a home monitoring preference. (Appendix E presents detailed regression analysis information).

## 4. Discussion

### 4.1. Theoretical Implications

Our analysis of the HOME^TA^ study shows that the relative preference for EEG home-monitoring compared to inpatient monitoring does not statistically significantly depend on participant type (patient or non-patient), age, gender, health status, graduation, or vocational qualification. Importantly, watching an introductory video about the use of a mobile EEG system necessary for home monitoring does positively influence an individual’s preference for choosing home monitoring over inpatient monitoring. This could lead us to assume that the provision of more information in advance regarding how to handle a medical device as part of a telemedical examination could help to increase acceptance. However, the most important predictor of preference regarding home monitoring is the behavioral intention to use this health care option, which is the final endogenous construct of our extended UTAUT model.

After confirmation of the UTAUT model’s reliability and validity, the analysis confirmed three significant direct and three significant indirect drivers of BI. Predictor PE is the strongest direct driver–in line with previous studies [71,72,73]–closely followed by EE. Effort expectancy (EE) has a similar influence on BI as expected benefit and improvement of health (PE), perhaps because the home-monitoring examination includes the autonomous use of an unknown medical device, which could prove more challenging than other telehealth services requiring only the use of desktop or mobile applications, for example, which might be more familiar. In this context, the level of trust that individuals have in user-friendly applications is even more important. The third direct predictor of BI is FC. According to our multigroup analysis, the belief that technical support will be available in case of problems during the performance of the home-monitoring examination has a stronger impact on BI for patients than for non-patients. Patients we recruited for our study are already familiar with the EEG home-monitoring system and, for this reason, are more willing to carry out EEG home-monitoring if they can count on support in the event of necessity. Finally, SI does not have an influence on BI, which is in line with a previous study of the acceptance of home telehealth services by older users [46]. Even though our study is not limited to elderly people, the opinions of peers, colleagues, and family are not found to be important when it comes to decisions of whether to use home monitoring or not. Considering that the UTAUT model was originally created for users’ acceptance of technology in a working environment [42] and not in a private space, as well as with the knowledge of previous studies affirming the impact of background situations on SI [46,74], the opinions of others do not seem to be a key decision-making criterium when it comes to pursuing health improvements.

The factors of CA, PS, and DC do have an influence on EE or PE, making them indirect BI drivers. The strongest indirect predictor is the absence of computer anxiety. In this context, our homemade examination includes the use of a mobile application installed on a provided tablet, which makes this aspect important for our analysis. Decreasing computer anxiety increases the belief in ease of use and results in the behavioral intention to use the home-monitoring system. DC and PS support the increase of ‘belief in an improvement of personal health’ directly and on BI indirectly. In summary, trust in the safety of personal health data and in doctors’ expertise do both have an indirect impact on BI, but a much lower one than decreasing computer anxiety.

An additional IPMA analysis clarifies that in total, the constructs EE, CA, and PE are most important in explaining the respondents BI to use EEG home-monitoring. However, this analysis also confirms that all three constructs have above-average status-quo, leaving only marginal potential for improvements. In contrast, although PS only have marginal impact on BI, this construct provides the highest potential for learning. Thus, proper communication of facts about data security should not be neglected.

Due to the lack of studies assessing the acceptance of EEG home-monitoring, we compare the results of the direct predictors in our study with those of two studies that investigate the acceptance of wearable healthcare devices [51,52]. In line with our results, both studies report PE to have a direct effect on BI. In contrast to the results reported here, in [51] EE and FC do not have any influence on BI. Regarding these differences, we assume the task complexity and the necessity of a patient’s commitment (when implementing the EEG home-monitoring) are possible causes of why we found EE and FC to have a stronger effect. Another difference to our results is that both studies report a positive effect of SI on BI. This difference might be explained—at least to some extent—by the device used. While the use of wearables depends on a high level of awareness within the group of (potential) customers, this awareness is not necessary for the use of a medical device such as the mobile EEG device used in our study. Here, the use of the medical device is based on a doctor’s prescription.

In terms of theoretical implications, we conclude from our study: (1) the preference for EEG home-monitoring compared to inpatient monitoring depends on the behavioral intention to use home monitoring and can be assessed by using an extended UTAUT model [46]. We support the conclusion of [46] who also found six relevant predictors in their study on older users’ home telehealth services acceptance behavior. More specifically, similar to [46], we (2) found three significant direct drivers (PE, EE, and FC) and three significant indirect drivers (DC, CA, and PS). The (3) insignificant effect of social influence is also in line with previous research and can be related to the origin of the UTAUT model. The model was originally developed to examine technology acceptance within organizations, e.g., companies. Here, it is plausible to assume that the opinion of the social environment, such as that of colleagues, has a major influence. However, since medical devices are prescribed by doctors, it is understandable that the influence of the social environment is less important. Therefore, future investigations can take this aspect into account.

### 4.2. Practical Implications

The current HOME^TA^ study is part of the *HOME* project, which aims to provide evidence of diagnostic and therapeutic yield (“change of management”) of EEG home-monitoring neurological outpatients [37]. To meet the key goals of the project, it was necessary to confirm the technical usability and efficacy of the new EEG device (see [38,40]) but also to demonstrate the feasibility and diagnostic/therapeutic yield of EEG home-monitoring neurological patients (see [39]). In order to establish EEG home-monitoring as a new health service [75] and, thus, to gain practical relevance, the patients’ acceptance of this new health service is of crucial importance. In this regard, the HOME^TA^ study results provide practical implications that have to be taken into consideration for the design and implementation of EEG home-monitoring as a standard alternative to inpatient monitoring. 

The influence of CA and EE on BI suggests that when patients decide to use an EEG home-monitoring system autonomously they have to feel comfortable and secure, without fear of failing. This situation could be achieved via a user-friendly system design and adequate training in advance. At the time of taking a decision for or against the home-based examination, patients could be shown a short video about the EEG system that, as demonstrated in our study, can have a positive effect on the decision-making process. That means, providing the necessary information at the right time and in a way that patients can understand easily is particularly important to gain acceptance for such a health service. In this regard, we agree with [46] that physicians have a special role in this context. As social agents, physicians have to promote this health service by prescribing the use of EEG home-monitoring (see [46] p. 29).

In addition, the impact of FC on BI suggests that available support in case of problems also plays an important role. While doctors’ expertise and data safety are of less importance in comparison to other factors, they still influence BI. For this reason, the home-monitoring concept should be aligned to suit, e.g., through the provision of sufficient information on the examination procedure and data security. According to our study, the opinions of peers, friends, and family do not influence decision-making processes on home-monitoring use.

Based on the current HOME^TA^ results and the results derived previously throughout the *HOME* project we summarize that EEG home-monitoring neurological patients can be well integrated into outpatient care. This new health service could be considered as an alternative for some cases of expensive inpatient monitoring, if the patients’ information needs are considered and if the positive effects on patients’ health are highlighted.

### 4.3. Limitations and Further Research

Despite these interesting findings, the study does have some limitations. For example, the survey relied upon an online structure (the home-monitoring system video should be shown randomly), largely for organizational reasons (use of an online panel and carried out during the COVID-19 pandemic). In taking this approach, although we drew participants from all age groups, we were unable to include people who partially or totally refused to embrace computer technology. Furthermore, with the study implementation occurring at the time of the COVID-19 pandemic, it is likely that participants may have felt a more positive attitude to telemedicine than previously. In addition, it remains to be seen whether attitudes may shift again once the pandemic is in the past, so future research might do well to focus on this. 

So far, the *HOME* project has mainly examined technical aspects (usability and feasibility of an EEG home-monitoring) and the perspective of patients (acceptance of an EEG home-monitoring). Future research should consider the perspective of physicians. For this purpose, the attitude of physicians to this health service could be analyzed in qualitative studies. At the same time, specific requirements from the physicians’ perspective can be examined in depth. Thus, the focus group research method could be applied in order to identify wishes, requirements and possible problems physicians may anticipate with an EEG home-monitoring of their patients.

## 5. Conclusions

The aim of the HOME^TA^ study was to examine neurological patients’ preferences regarding EEG home-monitoring compared to inpatient monitoring, and to gain a better understanding of the predictors behind the preferences. The HOME^TA^ results complement the previous results of the *HOME* project and, thus, contribute to evaluating EEG home-monitoring for neurological patients as a new health service and to demonstrating how this service can be integrated into outpatient care.

For this purpose, we used an extended version of the UTAUT model to assess factors considered relevant for developing an EEG home-monitoring concept that patients will accept as an alternative to inpatient monitoring. In addition to the factors incorporated in the extended UTAUT model, we considered several control variables, such as gender, age, and health status, to evaluate which factors have to be taken into consideration when developing a strategy to implement EEG home-monitoring as a new health service. In this regard, we recruited (1) 40 patients from the University Hospital in Magdeburg (Germany), and (2) 381 non-patients from an online panel provider. Approximately half of the total 421 participants were randomly assigned into either a video-condition or a no-video condition.

Our study shows behavioral intention (BI) to be the main driver behind preferences for home-monitoring examination, which is applicable for choosing an autonomous use of a medical device over inpatient monitoring. BI is the final endogenous construct of the UTAUT model for which we can confirm the validity in the context of EEG home-monitoring. The behavioral intention to use this approach depends on various views and beliefs, such as: (1) home-monitoring improves health, (2) the home-monitoring system is easy to use, (3) there will be technical support in case of issues during the home recording, (4) I am not afraid of using the home-monitoring system, (5) my health data are safe, and (6) the doctor is an expert. Meeting these expectations is a crucial task when creating and designing a home-monitoring concept, which can be achieved by implementing user-friendliness, patient training, and provision of comprehensive information covering the new medical care option.

## Figures and Tables

**Table 1:** Participants’ characteristics (N=421).

Characteristic	Patient/ Video (*n* = 15)	Patient/ No-Video (*n* = 25)	Non-Patient/Video (*n* = 200)	Non-Patient/No-Video (*n* = 181)	Full Sample (*n* = 421)
**age (F(3, 417) = 0.923; *p* = 0.430)**					
mean (SD)	44.33 (13.75)	51.76 (16.96)	48.73 (13.87)	49.61 (15.16)	49.13 (14.62)
**gender (Fisher’s exact *p* = 0.080)**					
male	9 (60.0%)	17 (68.0%)	103 (51.5%)	102 (56.4%)	231 (54.9%)
female	6 (40%)	7 (28.0%)	97 (48.5%)	79 (43.6%)	189 (44.9%)
divers	0 (0.0%)	1 (4.0%)	0 (0.0%)	0 (0.0%)	1 (0.2%)
Graduation * ( $X^{2}$ **(15) = 15.91; *p* = 0.388)**					
main school	1 (6.7%)	1 (4.2%)	23 (11.5%)	19 (10.5%)	44 (10.5%)
secondary school	1 (6.7%)	7 (29.2%)	23 (11.5%)	17 (9.4%)	48 (11.4%)
middle school	3 (20.0%)	2 (8.3%)	58 (29.0%)	53 (29.3%)	116 (27.6%)
university	10 (66.7%)	14 (58.3%)	93 (46.5%)	90 (49.7%)	207 (49.3%)
none	0 (0.0%)	0 (0.0%)	1 (0.5%)	0 (0.0%)	1 (0.2%)
prefer not to say	0 (0.0%)	0 (0.0%)	2 (1.0%)	2 (1.1%)	4 (1.0%)
**Vocational qualification (** $X^{2}$ **(27) = 32.25; *p* = 0.223)**					
apprenticeship	7 (46.7%)	8 (32.0%)	90 (45.0%)	83 (45.9%)	188 (44.7%)
professional school degree	1 (6.7%)	2 (8.0%)	30 (15.0%)	27 (14.9%)	60 (14.3%)
professional school degree (incl. administrative and engineer college degree)	1 (6.7%)	1 (4.0%)	9 (4.5%)	9 (5.0%)	20 (4.8%)
college degree	3 (30.0%)	2 (8.0%)	13 (6.5%)	7 (3.9%)	25 (5.9%)
Bachelor’s degree	0 (0.0%)	0 (0.0%)	17 (8.5%)	8 (4.4%)	25 (5.9%)
Master’s degree	0 (0.0%)	1 (4.0%)	10 (5.0%)	9 (5.0%)	20 (4.8%)
diploma	1 (6.7%)	6 (24.0%)	15 (7.5%)	18 (9.9%)	40 (9.5%)
promotion	0 (0.0%)	0 (0.0%)	3 (1.5%)	3 (1.7%)	6 (1.4%)
none	0 (0.0%)	3 (12.0%)	5 (2.5%)	10 (5.5%)	18 (4.3%)
prefer not to say	2 (13.3%)	2 (8.0%)	8 (4.0%)	7 (3.9%)	19 (4.5%)

* One observation missing within the group patient/no-video.

**Table 2:** Bootstrapping results of the UTAUT model.

Path	Total Effect *(t-Value/p-Value/[95% CI])*		Direct Effect *(t-Value/p-Value/[95% CI])*		Indirect Effect *(t-Value/p-Value/[95% CI])*	
DC → BI	0.065 (3.016/0.003/[0.028; 0.110])		-		0.065 (3.016/0.003/[0.028; 0.110])	
CA → BI	0.397 (8.533/0.000/[0.308; 0.489])		-		0.397 (8.533/0.000/[0.308; 0.489])	
PS → BI	0.142 (3.632/0.000/[0.066; 0.217])		0.047 (1.176/0.239/ [−0.031; 0.125])		0.095 (3.872/0.000/[0.050; 0.147])	
					via EE 0.018 (1.737/0.082/ [−0.001; 0.039])	via PE 0.066 (3.278/0.001/[0.030; 0.109])
PE → BI	0.348 (5.381/0.000/[0.223; 0.472])		0.348 (5.381/0.000/[0.223; 0.472])		-	
EE → BI	0.508 (9.549/0.000/[0.403; 0.611])		0.312 (5.091/0.000/[0.189; 0.431])		0.196 (4.921/0.000/[0.122; 0.276])	
FC → BI	0.184 (3.802/0.000/[0.090; 0.278])		0.184 (3.802/0.000/[0.090; 0.278])		-	
SI → BI	0.057 (1.126/0.260/ [−0.041; 0.160])		0.057 (1.126/0.260/ [−0.041; 0.160])		-	
EE → PE	0.562 (11.563/0.000/[0.461; 0.655])		0.562 (11.563/0.000/[0.461; 0.655])		-	
CA → EE	0.781 (24.808/0.000/[0.714; 0.837])		0.781 (24.808/0.000/[0.714; 0.837])		-	
PS → EE	0.056 (1.859/0.063/ [−0.004; 0.116])		0.056 (1.859/0.063/ [−0.004; 0.116])		-	
PS → PE	0.221 (5.296/0.000/ [0.140; 0.304])		0.189 (4.429/0.000/ [0.106; 0.273])		0.032 (1.800/0.072/ [−0.002; 0.067])	
DC → PE	0.184 (4.103/0.000/[0.100; 0.272])		0.184 (4.103/0.000/[0.100; 0.272])		-	
CA → PE	0.440 (9.884/0.000/[0.351; 0.525])		-		0.440 (9.884/0.000/[0.351; 0.525])	

BI indicates behavioral intention to use; CA, computer anxiety; DC, doctor’s opinion; EE, effort expectancy; FC, facilitating conditions; PE, performance expectancy; PS, perceived security; and SI, social influence.

**Table 3:** PLSpredict RMSE results of the UTAUT model.

Item	PLS-SEM RMSE	Linear Model RMSE
BI1	0.877	0.853
BI2	0.971	1.002
BI3	0.902	0.886
BI4	0.863	0.823
PE1	0.915	0.890
PE2	1.281	1.304
PE3	1.092	1.135
PE4	1.235	1.200
PE5	1.037	1.027
EE1	0.989	0.965
EE2	0.877	0.864
EE3	0.886	0.937
EE4	0.883	0.914

RMSE indicates the root mean squared error of prediction.

**Table 4:** Regression analysis among home monitoring preferences and various predictors.

Dependent Variable = Home Monitoring Preference		Model 1	Model 2	Model 3	Model 4
		b (t/p)	b (t/p)	b (t/p)	b (t/p)
	Intercept	5.147 (60.097/0.000)	5.398 (13.223/0.000)	5.693 (8.935/0.000)	5.391 (8.500/0.000)
Main variable	Behavioral intention score	0.973 (11.364/0.000)	1.015 (11.605/0.000)	1.012 (11.575/0.000)	1.007 (11.563/0.000)
Health	Physical functioning		−0.002 (−0.294/0.769)	−0.003 (−0.483/0.629)	−0.004 (−0.705/0.481)
	Physical health		0.004 (1.124/0.262)	0.004 (0.929 /0.354)	0.004 (1.054/0.292)
	Emotional problems		0.004 (1.184/0.237)	0.005 (1.332/0.184)	0.005 (1.392/0.165)
	Energy/fatigue		−0.003 (−0.450/0.653)	−0.001 (−0.122/0.903)	−0.002 (−0.274/0.784)
	Emotional well-being		−0.008 (−0.920/0.358)	−0.006 (−0.775/0.439)	−0.006 (−0.726/0.469)
	Social functioning		−0.010 (−1.935/0.054)	−0.010 (−1.919/0.056)	−0.010 (−1.828/0.068)
	Pain		0.002 (0.361/0.752)	0.003 (0.473/0.636)	0.003 (0.553/0.581)
	General health		0.009 (1.350/0.178)	0.006 (0.850/0.396)	0.005 (0.811/0.418)
	Health change		0.003 (0.556/0.579)	0.002 (0.363/0.716)	0.002 (0.343/0.732)
Demographics	Age			−0.009 (−1.337/0.182)	−0.008 (-1.277/0.202)
	Gender (0 = male, 1 = female)			0.177 (1.014/0.311)	0.166 (0.952/0.342)
Conditions	Participant type				0.331 (1.107/0.269)
	Video (0 = no, 1 = yes)				0.466 (2.714/0.007)
Summary	Observations	421	421	420	420
	$R^{2}$	0.236	0.259	0.264	0.278
	Adjusted $R^{2}$	0.234	0.240	0.242	0.253

Note. Model 3 and Model 4 exclude the one participant that identifies as “divers”. Higher values on the dependent variable represent a higher relative preference for home monitoring compared to inpatient monitoring.

**Figure A1:**
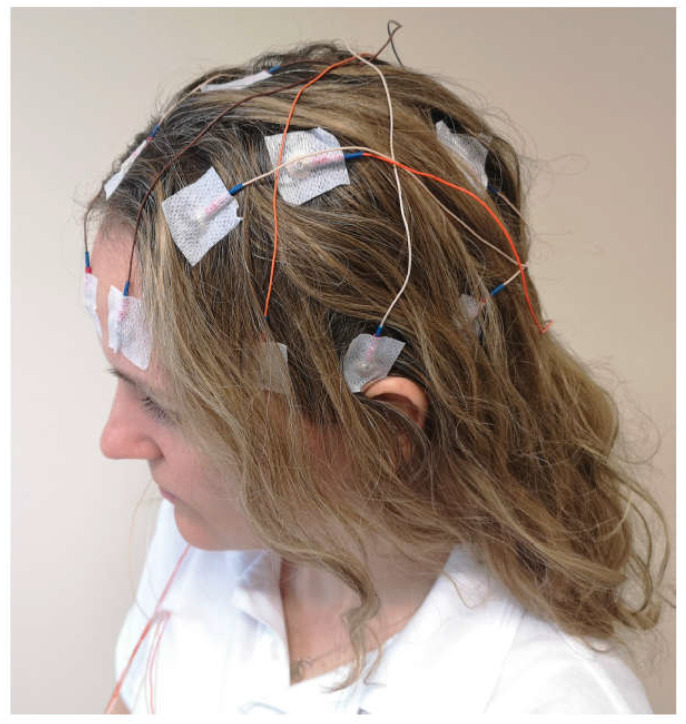
Option 1: EEG Inpatient Monitoring.

**Figure A2:**
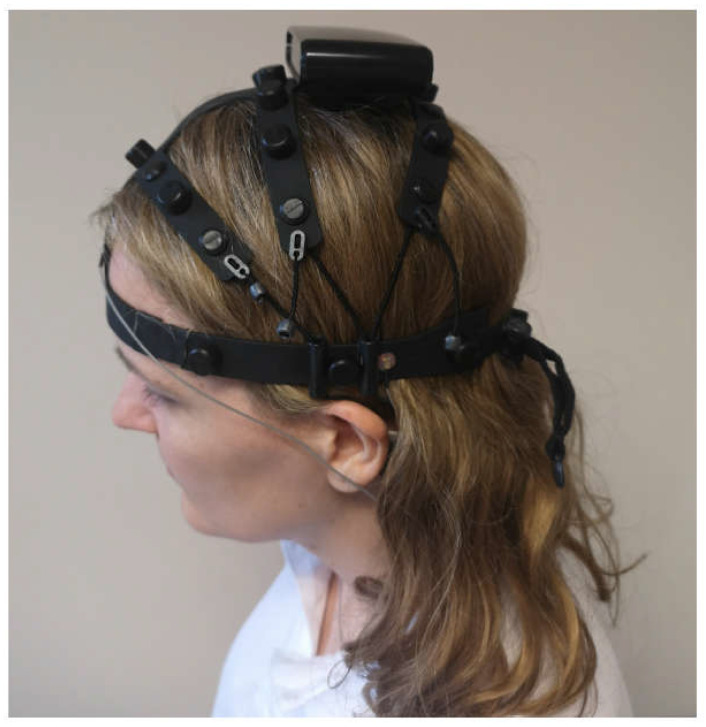
Option 2: EEG Home-Monitoring.

## Data Availability

The corresponding datasets of this study are available from the corresponding author upon reasonable request.

## Appendix A. Survey Scenario

### Please Imagine the Following Situation

You are sitting in the garden with your friends on a warm and sunny day. You are thirsty and get up to get something to drink. The next thing you know, you wake up lying on the ground without remembering what happened. Your friends tell you that you suddenly collapsed and were unresponsive for a short time. Although you feel fine again and have suffered no injuries due to a soft landing on the grass, the next day you go to your family doctor, who refers you to a neurologist (specialist in neurology). Based on your history, your neurologist recommends that you have electroencephalography (EEG) examination. During this examination, electrodes are placed on your scalp to record the electrical impulses of brain activity. There is the possibility of performing this examination over a short period of time (approximately 20 min) or over a longer period (several hours). In your case, the neurologist suggests a long-term measurement in order to be able to evaluate for a longer period of time. This long-term EEG can be performed either in a hospital or in your home.

## Appendix B. Presentation of Inpatient Monitoring and Home-Monitoring Options

### Appendix B.1. Option 1: EEG Inpatient Monitoring

You are hospitalized and admitted for 24 h. The examination proceeds as follows: A medical-technical assistant places the electrodes on your scalp for long-term EEG measurement and then starts the EEG recording. You are not allowed to attach or detach the electrodes by yourself.

The wearing time of the electrodes depends on your neurologist’s instructions. You can move around while wearing the electrodes, but should only engage in moderate activities (i.e., no sports, etc.). At the end of the wearing time, the medical-technical assistant stops the recording and removes the electrodes. Following your hospital discharge, you will need to attend a meeting at your neurologist’s office where the EEG results will be discussed with you.

### Appendix B.2. Option 2: EEG Home-Monitoring

You are at your home and a nursing service supplies you with a mobile EEG system with accessories. The nursing service also provides you with detailed instructions on how to place and remove the EEG cap autonomously and how to start the EEG recording. The electrodes are already positioned within the cap and, with the help of a supplied tablet (mini-computer), you can start the recording. After the nursing service has said goodbye, you can put on the cap by yourself for the long-term EEG measurement and start the recording with the tablet (mini-computer). While you are wearing the mobile EEG cap, you can move around, but should only engage in moderate activities (i.e., no sports, etc.). The recorded EEG data is sent to the server of your neurologist in an encrypted way via an included router. Compliance with all data privacy directives and safety regulations is warranted. Your neurologist informs you about the necessary wearing time of the mobile EEG cap. After completion of the wearing time, you stop the EEG recording and then remove the mobile EEG cap by yourself. In agreement with you, the nursing service collects the mobile EEG system from your home. Your neurologist will discuss the EEG results with you during a follow-up appointment in the doctor’s office.

## Appendix C. Measurement Information for the Extended UTAUT Model

The SEM quality assessment starts with evaluating the model’s reliability. To begin with, internal consistency reliability holds with all Cronbach’s α values higher than 0.7 (Table A1). Almost all outer loadings exceed 0.7, ensuring indicator reliability. Additionally, although one item of DC possesses an outer loading of 0.566, convergent validity holds for all constructs with an average variance extracted (AVE) above 0.5.

**Table A1:** Item wording and PLS measurement model results.

Construct	Item	Loading	English Wording	German Wording
**Performance Expectancy (PE)**–the degree to which an individual believes that using EEG home-monitoring will help him or her increase their health performance/quality				
*AVE = 0.702* *α = 0.894* *C.R. = 0.922*	PE1	0.816	I find that using EEG home-monitoring would be helpful in monitoring my health.	Ich denke, dass die Durchführung eines EEG-Home-Monitorings bei der Diagnostik meines Krankheitsbildes und der Überwachung meiner Gesundheit hilfreich ist.
	PE2	0.811	I find that using EEG home-monitoring would make me feel safer in my daily life.	Ich denke, dass ich mich durch die Durchführung eines EEG-Home-Monitorings sicherer fühle, was meine Gesundheit betrifft.
	PE3	0.838	EEG home-monitoring could enhance the level of convenience in accessing medical care services.	Die Möglichkeit, eine EEG-Untersuchung zu Hause durchführen zu können (EEG-Home-Monitoring), könnte den Zugang zur medizinischen Versorgung erleichtern.
	PE4	0.829	EEG home-monitoring could enhance the quality of my life.	Das EEG-Home-Monitoring als Untersuchung in der Häuslichkeit könnte meine Lebensqualität verbessern.
	PE5	0.894	Overall, I find that EEG home-monitoring would be highly useful.	Insgesamt finde ich das EEG-Home-Monitoring sehr nützlich.
**Effort Expectancy (EE)**–the degree of ease associated with the use of EEG home-monitoring				
*AVE = 0.819* *α = 0.926* *C.R. = 0.948*	EE1	0.896	I find that using EEG home-monitoring would be simple.	Ich finde, dass die Durchführung des EEG-Home-Monitorings einfach wäre.
	EE2	0.928	I find that using EEG home-monitoring would be easy to learn.	Ich finde, dass die Durchführung des EEG-Home-Monitorings leicht zu erlernen wäre.
	EE3	0.920	I find that EEG home-monitoring would be easily understandable and clear for me.	Ich finde, dass das EEG-Home-Monitoring für mich leicht verständlich und klar wäre.
	EE4	0.876	Overall, I find that using EEG home-monitoring would be convenient.	Insgesamt finde ich die Verwendung des EEG-Home-Monitorings praktisch.
**Social Influence (SI)**–influence of peers and colleagues’ opinions				
*AVE = 0.808* *α = 0.881* *C.R. = 0.927*	SI1	0.870	Peers and colleagues would support me in using EEG home-monitoring.	Gleichaltrige und Kollegen würden mich bei der Entscheidung, das EEG-Home-Monitoring durchzuführen, unterstützen.
	SI2	0.915	People who influence my behavior would support my use of EEG home-monitoring.	Menschen, die mich beeinflussen, würden mich bei der Entscheidung, das EEG-Home-Monitoring durchzuführen, unterstützen.
	SI3	0.911	People who are important to me would support my use of EEG home-monitoring.	Menschen, die mir wichtig sind, würden mich bei der Entscheidung, das EEG-Home-Monitoring zu durchzuführen, unterstützen.
**Facilitating Conditions (FC)**–technical support for using EEG home-monitoring				
*AVE = 0.741* *α = 0.824* *C.R. = 0.895*	FC1	0.870	I believe that guidance will be available to me when deciding whether to use EEG home-monitoring.	Ich gehe davon aus, dass ich bei meinem Neurologen ein Aufklärungsgespräch erhalten werde, wenn ich entscheiden muss, ob ich das EEG-Home-Monitoring durchführen möchte.
	FC2	0.912	I believe that specialized instructions concerning the use of EEG home-monitoring will be available to me.	Ich gehe davon aus, dass mir spezielle Anweisungen zur Verwendung des EEG-Home- Monitorings zur Verfügung stehen.
	FC3	0.796	I believe that specific persons (or a group) will be available for assistance with EEG home-monitoring difficulties (e.g., nursing service or a call center).	Unabhängig von der Einweisung durch den Pflegedienst gehe ich davon aus, dass ich während der Nutzung des EEG-Home- Monitorings Unterstützung bei Anwendungsschwierigkeiten erhalten werde (z. B. durch den Pflegedienst oder ein Callcenter).
**Computer Anxiety (CA)**–anxiety concerning the use of EEG home-monitoring option				
*AVE = 0.674* *α = 0.838* *C.R. = 0.892*	CA1	0.824	Anyone can learn to use a mobile EEG cap if they are patient and motivated.	Jeder kann lernen, eine mobile EEG-Haube zu verwenden, wenn er geduldig und motiviert ist.
	CA2	0.855	I do not hesitate to use a mobile EEG cap for fear of making mistakes.	Ich zögere nicht, eine mobile EEG-Haube zu verwenden, weil ich keine Angst habe, Fehler zu machen.
	CA3	0.855	If given the opportunity, I would like to learn about and use the mobile EEG cap.	Wenn ich die Gelegenheit dazu hätte, würde ich gerne die mobile EEG-Haube kennenlernen und nutzen.
	CA4	0.744	I feel that computers are necessary tools in both educational and work settings.	Ich bin der Meinung, dass Computertechnik in der Medizin notwendiges Werkzeug ist.
**Perceived Security (PS)**–the degree to which using information technology enables the administration of personal health information				
*AVE = 0.869* *α = 0.950* *C.R. = 0.964*	PS1	0.920	I would feel secure sending personal health information using the Internet and computers.	Ich würde mich sicher fühlen, persönliche Gesundheitsinformationen über das Internet zu senden.
	PS2	0.916	The Internet offers a secure means through which to send sensitive personal information.	Das Internet ist ein sicheres Medium, um vertrauliche persönliche Informationen zu senden.
	PS3	0.950	I would feel totally safe providing sensitive personal information about myself over the Internet.	Ich würde mich absolut sicher fühlen, sensible persönliche Informationen über das Internet bereitzustellen.
	PS4	0.943	Overall, using the EEG cap and an Internet connection is a safe way to transmit sensitive personal health information.	Insgesamt ist die Verwendung der EEG-Haube und einer Internetverbindung eine sichere Möglichkeit, vertrauliche persönliche Gesundheitsinformationen zu übertragen.
**Doctor’s Opinion (DC)**–doctor’s expert power influence				
*AVE = 0.683* *α = 0.919* *C.R. = 0.937*	DC1	0.877	I trust my doctor’s judgment.	Ich vertraue dem Urteil meines Arztes.
	DC2	0.907	The doctor’s expertise makes him/her more likely to be right.	Aufgrund des medizinischen Fachwissens des Arztes ist es wahrscheinlicher, dass er/sie recht hat.
	DC3	0.906	The doctor has a lot of experience and usually knows best.	Der Arzt hat viel Erfahrung und weiß es normalerweise am besten.
	DC4	0.871	The doctor’s knowledge usually makes him/her right.	Durch sein Wissen hat der Arzt für gewöhnlich recht.
	DC5	0.867	I trust my doctor’s judgment about the use of EEG home-monitoring.	Ich vertraue dem Urteil meines Arztes, was den Einsatz des EEG-Home-Monitorings betrifft.
	DC6	0.566	In the case of deciding to use EEG home-monitoring, I don’t know as much about what is required as the doctor does.	Wenn ich mich für das EEG-Home-Monitoring entscheide, kenne ich mich damit nicht so gut aus wie der Arzt.
	DC7	0.734	Doctors are intelligent.	Ärzte sind klug.
**Behavioral Intention to Use (BI)**–the degree to which an individual intends to use EEG home-monitoring				
*AVE = 0.869* *α = 0.950* *C.R. = 0.964*	BI1	0.913	Assuming there was a medical need to perform EEG home-monitoring, I would use it.	Angenommen es bestünde die medizinische Notwendigkeit, das EEG-Home-Monitoring durchzuführen, würde ich es verwenden.
	BI2	0.928	I assume that in the future I would regularly use EEG home-monitoring if it was medically necessary.	Ich gehe davon aus, dass ich bei Notwendigkeit in Zukunft regelmäßig das EEG-Home-Monitoring verwenden würde.
	BI3	0.937	I intend to use EEG home-monitoring in the future if medical necessity exists.	Ich beabsichtige, in Zukunft das EEG-Home-Monitoring zu verwenden, falls die Notwendigkeit besteht.
	BI4	0.950	Providing I had access to EEG home-monitoring, I would use the services when needed.	Wenn ich Zugang zur Verwendung des EEG-Home-Monitorings hätte, würde ich die Dienste bei Notwendigkeit nutzen.

Note. All items are scored on a 7-point Likert scale and range from 1 (totally disagree) to 7 (totally agree). AVE = average variance extracted, α = Cronbach’s Alpha, C.R. = composite reliability.

Next, we use the Fornell–Larcker criterion [76] to assess discriminant validity. Table A2 points out that the square roots of the average variance extracted on the diagonal (marked in gray) are higher for each of the constructs than the inter-construct correlations to any other latent construct (presented in the column below the diagonal values). Furthermore, Heterotrait-Monotrait Ratios in the upper triangular matrix confirm discriminant validity for the reflectively measured constructs [77]. Specifically, the 95% CIs do not include 1 (HTMT_inference_ < 1). For the sake of completeness, Table A3 presents variance inflation factors (VIF) regarding each endogenous construct in the model. All values are below 5, thus, no issues with multicollinearity among exogenous constructs can be identified.

**Table A2:** Discriminant validity measurement.

Construct	Behavioral Intention (BI)	Computer Anxiety (CA)	Doctor’s Opinion (DC)	Effort Expectancy (EE)	Facilitating Conditions (FC)	Performance Expectancy (PE)	Perceived Security (PS)	Social Influence (SI)
Behavioral intention (BI)	0.932	[0.799; 0.917]	[0.444; 0.638]	[0.706; 0.825]	[0.581; 0.758]	[0.714; 0.847]	[0.322; 0.489]	[0.488; 0.683]
Computer anxiety (CA)	0.771	0.821	[0.401; 0.637]	[0.836; 0.961]	[0.705; 0.856]	[0.718; 0.882]	[0.362; 0.533]	[0.591; 0.791]
Doctor’s opinion (DC)	0.520	0.467	0.826	[0.345; 0.560]	[0.471; 0.708]	[0.421; 0.623]	[0.245; 0.425]	[0.356; 0.552]
Effort expectancy (EE)	0.722	0.803	0.432	0.905	[0.582; 0.755]	[0.705; 0.843]	[0.309; 0.476]	[0.557; 0.731]
Facilitating conditions (FC)	0.602	0.647	0.534	0.595	0.861	[0.537; 0.729]	[0.192; 0.367]	[0.460; 0.657]
Performance expectancy (PE)	0.725	0.702	0.486	0.712	0.553	0.838	[0.410; 0.573]	[0.521; 0.717]
Perceived security (PS)	0.394	0.410	0.319	0.376	0.255	0.461	0.932	[0.331; 0.524]
Social influence (SI)	0.542	0.602	0.417	0.586	0.479	0.561	0.398	0.899

Notes: Gray main diagonal ($\sqrt[{2}]{AVE}$) and lower triangular matrix (Pearson correlation) present Fornell–Larcker criterion. Upper triangular matrix presents the Heterotrait-Monotrait Ratio of correlations (95% CIs).

**Table A3:** Collinearity Check.

Variance Inflation Factors (VIF)	Behavioral Intention (BI)	Effort Expectancy (EE)	Performance Expectancy (PE)
Computer anxiety (CA)		1.203	
Doctor’s opinion (DC)			1.274
Effort expectancy (EE)	2.470		1.333
Facilitating conditions (FC)	1.678		
Performance expectancy (PE)	2.405		
Perceived security (PS)	1.322	1.203	1.207
Social influence (SI)	1.721		

## Appendix D. Items Wording and Scale Quality Assessment of 36-Short Form Health Survey

**English Wording**	**German Wording**
**Physical functioning** *α = 0.946* (0 = yes, limited a lot; 50 = yes, limited a little; 100 = no, not limited at all)	
The following items are about activities you might carry out during a typical day. Does your health now limit you in these activities? If so, how much?	Im Folgenden sind einige Tätigkeiten beschrieben, die Sie vielleicht an einem normalen Tag ausüben. Sind Sie durch Ihren derzeitigen Gesundheitszustand bei diesen Tätigkeiten eingeschränkt? Wenn ja, wie stark?
vigorous activities, such as running, lifting heavy objects, participating in strenuous sports	anstrengende Tätigkeiten, z.B. schnell laufen
moderate activities, such as moving a table, pushing a vacuum cleaner, bowling, or playing golf	mittelschwere Tätigkeiten, z.B. einen Tisch verschieben, staubsaugen, kegeln, Golf spielen
lifting or carrying groceries	Einkaufstaschen heben oder tragen
climbing several flights of stairs	mehrere Treppenabsätze steigen
climbing one flight of stairs	einen Treppenabsatz steigen
bending, kneeling, or stooping	sich beugen, knien, bücken
walking more than a mile	mehr als 1 Kilometer zu Fuß gehen
walking several blocks	mehrere Straßenkreuzungen weit zu Fuß gehen
walking one block	eine Straßenkreuzung weit zu Fuß gehen
bathing or dressing yourself	sich baden oder anziehen
**Physical health** *α = 0.888* (0 = yes, 100 = no)	
During the past 4 weeks, have you experienced any of the following problems with your work or other regular daily activities as a result of your physical health?	Hatten Sie in den vergangenen 4 Wochen aufgrund Ihrer körperlichen Gesundheit irgendwelche Schwierigkeiten bei der Arbeit oder anderen alltäglichen Tätigkeiten im Beruf bzw. zu Hause?
cut down the amount of time you spent on work or other activities	Ich konnte nicht so lange wie üblich tätig sein.
accomplished less than you would like	Ich habe weniger geschafft als ich wollte.
were limited in the kind of work or other activities	Ich konnte nur bestimmte Dinge tun.
had difficulty performing the work or other activities (for example, it took extra effort)	Ich hatte Schwierigkeiten bei der Ausführung.
**Emotional problems** *α = 0.909* (0 = yes, 100 = no)	
During the past 4 weeks, have you experienced any of the following problems with your work or other regular daily activities as a result of any emotional problems (such as feeling depressed or anxious)?	Hatten Sie in den vergangenen 4 Wochen aufgrund seelischer Probleme irgendwelche Schwierigkeiten bei der Arbeit oder anderen alltäglichen Tätigkeiten im Beruf bzw. zu Hause (z.B., weil Sie sich niedergeschlagen oder ängstlich fühlten)?
cut down the amount of time you spent on work or other activities	Ich konnte nicht so lange wie üblich tätig sein
accomplished less than you would like	Ich habe weniger geschafft als ich wollte.
didn’t complete work or other activities as carefully as usual	Ich konnte nicht so sorgfältig wie üblich arbeiten.
**Energy/fatigue** *α = 0.873* (100 = all of the time, 80 = most of the time, 60 = a good portion of the time, 40 = some of the time, 20 = a little of the time, 0 = none of the time)	
These questions are about how you feel and how things have been with you during the past four weeks. For each question, please give the one answer that comes closest to the way you have been feeling. How much of the time during the past four weeks...	In diesen Fragen geht es darum, wie Sie sich fühlen und wie es Ihnen in den vergangenen 4 Wochen gegangen ist. Wie oft waren Sie in den vergangenen 4 Wochen…
did you feel full of pep?	...voller Schwung?
did you have a lot of energy?	...voller Energie?
did you feel worn out? (R)	...erschöpft? (R)
did you feel tired? (R)	...müde? (R)
**Emotional well-being** *α = 0.882* (100 = none of the time, 80 = a little of the time, 60 = some of the time, 40 = a good portion of the time, 20 = most of the time, 0 = all of the time)	
These questions are about how you feel and how things have been with you during the past four weeks. For each question, please give the one answer that comes closest to the way you have been feeling. How much of the time during the past four weeks...	In diesen Fragen geht es darum, wie Sie sich fühlen und wie es Ihnen in den vergangenen 4 Wochen gegangen ist. Wie oft waren Sie in den vergangenen 4 Wochen…
have you felt like a very nervous person?	...sehr nervös?
have you felt so down in the dumps that nothing could cheer you up?	so niedergeschlagen, dass Sie nichts aufheitern konnte?
have you felt calm and peaceful? (R)	...ruhig und gelassen? (R)
have you felt downhearted and blue?	...entmutigt und traurig?
have you been a happy person? (R)	... glücklich? (R)
**Social functioning** *α = 0.895* (Item 1: 100 = not at all, 75 = a little bit, 50 = moderately, 25 = quite a bit, 0 = extremely. Item 2: 0 = all of the time, 25 = most of the time, 50 = some of the time, 75 = a little bit of the time, 100 = none of the time)	
During the past four weeks, to what extent have your physical health or emotional problems interfered with your normal social activities with family, friends, neighbors, or groups?	Wie sehr haben Ihre körperliche Gesundheit oder seelischen Probleme in den vergangenen 4 Wochen Ihre normalen Kontakte zu Familienangehörigen, Freunden, Nachbarn oder zum Bekanntenkreis beeinträchtigt?
During the past four weeks, how much of the time has your physical health or emotional problems interfered with your social activities (like visiting friends, relatives, etc.)? (R)	Wie häufig haben Ihre körperliche Gesundheit oder seelischen Probleme in den vergangenen 4 Wochen Ihre Kontakte zu anderen Menschen (Besuche bei Freunden, Verwandten usw.) beeinträchtigt?
**Pain***α = 0.903* (Item 1: 100 = none, 80 = very mild, 60 = mild, 40 = moderate, 20 = severe, 0 = very severe. Item 2: 100 = not at all, 75 = a little bit, 50 = moderately, 25 = quite a bit, 0 = extremely)	
How much bodily pain have you had during the past four weeks?	Wie stark waren Ihre Schmerzen in den vergangenen 4 Wochen?
During the past four weeks, how much did pain interfere with your normal work (including both work outside the home and housework)?	Inwieweit haben die Schmerzen Sie in den vergangenen 4 Wochen bei der Ausübung Ihrer Alltagstätigkeiten zu Hause und im Beruf behindert?
**General health** *α = 0.812*	
(Item 1: 100 = excellent, 75 = very good, 50 = good, 25 = fair, 0 = poor. Item 2–5: 100 = definitely true, 75 = mostly true, 50 = don’t know, 25 = mostly false, 0 = definitely false)	
In general, would you say that your health is:	Wie würden Sie Ihren Gesundheitszustand im Allgemeinen beschreiben?
I seem to get sick a little easier than other people (R)	Ich scheine etwas leichter als andere krank zu werden.
I am as healthy as anybody I know	Ich bin genauso gesund wie alle anderen, die ich kenne.
I expect my health to get worse (R)	Ich erwarte, dass meine Gesundheit nachlässt.
My health is excellent	Ich erfreue mich ausgezeichneter Gesundheit.
**Health change** (100 = Much better now than one year ago, 75 = Somewhat better now than one year ago, 50 = Approximately the same, 25 = Somewhat worse now than one year ago, 0 = Much worse now than one year ago)	
Compared to one year ago, how would you rate your health in general now?	Im Vergleich zum vergangenen Jahr, wie würden Sie Ihren derzeitigen Gesundheitszustand beschreiben?

## Appendix E. Detailed Information about Regression Analysis

**Model 1**	B	Std. Error	Beta	t	*p*	CI	
**(Constant)**	5.147	0.086		60.097	0.000	4.979	5.316
**BI score**	**0.973**	**0.086**	**0.485**	**11.364**	**0.000**	**0.805**	**1.142**
**Model 2**	B	Std. Error	Beta	t	*p*	CI	
(Constant)	5.398	0.408		13.223	0.000	4.596	6.201
**BI score**	**1.015**	**0.087**	**0.506**	**11.605**	**0.000**	**0.843**	**1.187**
Physical functioning	−0.002	0.005	−0.019	−0.294	0.769	−0.012	0.009
Physical health	0.004	0.004	0.082	1.124	0.262	−0.003	0.012
Emotional problems	0.004	0.003	0.078	1.184	0.237	−0.003	0.011
Energy /fatigue	−0.003	0.007	−0.035	−0.450	0.653	−0.017	0.011
Emotional well-being	−0.008	0.008	−0.078	−0.920	0.358	−0.024	0.009
Social functioning	−0.010	0.005	−0.135	−1.935	0.054	−0.021	0.000
Pain	0.002	0.005	0.022	0.316	0.752	−0.009	0.012
General health	0.009	0.006	0.092	1.350	0.178	−0.004	0.022
Health change	0.003	0.005	0.026	0.556	0.579	−0.007	0.013
**Model 3**	B	Std. Error	Beta	t	*p*	CI	
(Constant)	5.636	0.631		8.935	0.000	4.396	6.877
**BI score**	**1.012**	**0.087**	**0.505**	**11.575**	**0.000**	**0.840**	**1.184**
Physical functioning	−0.003	0.006	−0.032	−0.483	0.629	−0.014	0.008
Physical health	0.004	0.004	0.068	0.929	0.354	−0.004	0.011
Emotional problems	0.005	0.003	0.088	1.332	0.184	−0.002	0.011
Energy/fatigue	−0.001	0.007	−0.010	−0.122	0.903	−0.015	0.013
Emotional well-being	−0.006	0.008	−0.066	−0.775	0.439	−0.023	0.010
Social functioning	−0.010	0.005	−0.134	−1.919	0.056	−0.021	0.000
Pain	0.003	0.005	0.033	0.473	0.636	−0.008	0.013
General health	0.006	0.007	0.059	0.850	0.396	−0.007	0.019
Health change	0.002	0.005	0.017	0.363	0.716	−0.008	0.012
Age	−0.009	0.007	−0.064	−1.337	0.182	−0.022	0.004
Gender	0.177	0.174	0.044	1.014	0.311	−0.166	0.520
**Model 4**	B	Std. Error	Beta	t	*p*	CI	
(Constant)	5391	0.634		8500	0	4144	6637
**BI score**	**1007**	**0.087**	**0.503**	**11,563**	**0**	**0.835**	**1178**
Physical functioning	−0.004	0.005	−0.046	−0.705	0.481	−0.015	0.007
Physical health	0.004	0.004	0.077	1054	0.292	−0.004	0.012
Emotional problems	0.005	0.003	0.092	1392	0.165	−0.002	0.012
Energy/fatigue	−0.002	0.007	−0.021	−0.274	0.784	−0.016	0.012
Emotional well-being	−0.006	0.008	−0.061	−0.726	0.469	−0.022	0.01
Social functioning	−0.01	0.005	−0.126	−1828	0.068	−0.02	0.001
Pain	0.003	0.005	0.038	0.553	0.581	−0.007	0.013
General health	0.005	0.007	0.056	0.811	0.418	−0.008	0.018
Health change	0.002	0.005	0.016	0.343	0.732	−0.008	0.012
Age	−0.008	0.007	−0.061	−1277	0.202	−0.021	0.005
Gender	0.166	0.174	0.041	0.952	0.342	−0.176	0.508
Participant type	0.331	0.299	0.048	1107	0.269	−0.257	0.919
**Video**	**0.466**	**0.172**	**0.116**	**2714**	**0.007**	**0.128**	**0.803**
